# A Combined Systemic Strategy for Overcoming Cisplatin Resistance in Head and Neck Cancer: From Target Identification to Drug Discovery

**DOI:** 10.3390/cancers12113482

**Published:** 2020-11-23

**Authors:** Yin-Ju Chen, Guo-Rung You, Meng-Yu Lai, Long-Sheng Lu, Chang-Yu Chen, Lai-Lei Ting, Hsin-Lun Lee, Yuzuka Kanno, Jeng-Fong Chiou, Ann-Joy Cheng

**Affiliations:** 1 Graduate Institute of Biomedical Materials and Tissue Engineering, College of Biomedical Engineering, Taipei Medical University, Taipei 11031, Taiwan; yjchen1113@tmu.edu.tw (Y.-J.C.); lslu@tmu.edu.tw (L.-S.L.); 2 International Ph.D. Program in Biomedical Engineering, College of Biomedical Engineering, Taipei Medical University, Taipei 11031, Taiwan; 3 Department of Radiation Oncology, Taipei Medical University Hospital, Taipei 11031, Taiwan; 971010@h.tmu.edu.tw (L.-L.T.); b001089024@tmu.edu.tw (H.-L.L.); solomanc@tmu.edu.tw (J.-F.C.); 4 TMU Research Center of Cancer Translational Medicine, Taipei Medical University, Taipei 11031, Taiwan; 5 Department of Medical Biotechnology, Medical College, Chang Gung University, Taoyuan 33302, Taiwan; d000015193@cgu.edu.tw (G.-R.Y.); katefishlai@chopharma.com.tw (M.-Y.L.); 6 Graduate Institute of Biomedical Sciences, College of Medicine, Chang Gung University, Taoyuan 33302, Taiwan; 7 Division of Molecular Regulation of Inflammatory and Immune Disease, Research Institute for Biomedical Sciences, Tokyo University of Science, Chiba 278-0022, Japan; alexchen@m.u-tokyo.ac.jp (C.-Y.C.); 3B17026@ed.tus.ac.jp (Y.K.); 8 Graduate School of Medicine, The University of Tokyo, Tokyo 113-8654, Japan; 9 Department of Radiology, School of Medicine, College of Medicine, Taipei Medical University, Taipei 11031, Taiwan; 10 Taipei Cancer Center, Taipei Medical University, Taipei 11031, Taiwan; 11 Department of Medicinal and Life Sciences, Faculty of Pharmaceutical Sciences, Tokyo University of Science, Chiba 278-0022, Japan; 12 Department of Radiation Oncology, Chang Gung Memorial Hospital-Linkou, Taoyuan 33305, Taiwan

**Keywords:** cisplatin resistance, head and neck cancer, SPC25, celastrol, mitotic division, transcriptome

## Abstract

**Simple Summary:**

The efficiency of cisplatin is limited by drug resistance in head–neck cancer (HNC) patients. In this study, we established a cisplatin resistance (CR) cell model, generated CR related transcriptome profiling, and combined application of bioinformatics methodology to discover a possible way to overcome CR. Analysis of the functional pathway revealed that mitotic division is a novel mechanism significantly contributing to CR. Spindle pole body component 25 (SPC25), a kinetochore protein, was overexpressed in CR cells and significantly correlated with worse HNC patient survival. The silencing of SPC25 increased cisplatin sensitivity and reduced cancer stemness property. Integration of CR transcriptome profiling and drug database discovered a natural extract compound, celastrol, possessing a potent cytotoxic effect in CR cells to reverse CR. Thus, we combined systemic strategies to demonstrated that a novel biological process (mitotic cell division), a hub gene (SPC25), and a natural compound (celastrol) as novel strategies for the treatment of refractory HNC.

**Abstract:**

Cisplatin is the first-line chemotherapy agent for head and neck cancer (HNC), but its therapeutic effects are hampered by its resistance. In this study, we employed systemic strategies to overcome cisplatin resistance (CR) in HNC. CR cells derived from isogenic HNC cell lines were generated. The CR related hub genes, functional mechanisms, and the sensitizing candidates were globally investigated by transcriptomic and bioinformatic analyses. Clinically, the prognostic significance was assessed by the Kaplan–Meier method. Cellular and molecular techniques, including cell viability assay, tumorsphere formation assay, RT-qPCR, and immunoblot, were used. Results showed that these CR cells possessed highly invasive and stem-like properties. A total of 647 molecules was identified, and the mitotic division exhibited a novel functional mechanism significantly related to CR. A panel of signature molecules, MSRB3, RHEB, ULBP1, and spindle pole body component 25 (SPC25), was found to correlate with poor prognosis in HNC patients. SPC25 was further shown as a prominent molecule, which markedly suppressed cancer stemness and attenuated CR after silencing. Celastrol, a nature extract compound, was demonstrated to effectively inhibit SPC25 expression and reverse CR phenotype. In conclusion, the development of SPC25 inhibitors, such as the application of celastrol, maybe a novel strategy to sensitize cisplatin for the treatment of refractory HNC.

## 1. Introduction

Globally, the incidence of head and neck cancer (HNC) has continued to rise to approximately 11% in 2018 [1], with over 1.9 million individuals diagnosed with HNC at that time. In clinical practice, cisplatin is considered the standard therapy for HNC patients. Cisplatin is a platinum-based chemotherapy drug that comprises a heavy metal complex with a central platinum atom surrounded by two chlorine atoms and two ammonia molecules. The cytotoxic effect of cisplatin involves its reaction with DNA forming inter-strand, and intra-strand cross-linking that inhibits DNA replication and leads to cell apoptosis [2,3,4]. Some patients had good treatment outcomes after they were administered a high-dose of cisplatin (100 mg per square meter of body surface area intravenously every 21 days for three cycles); however, recurrence still develops in more than 65% of the HNC patients [5], which suggests the necessity for understanding the mechanisms of cisplatin resistance to explore a new approach to improve therapeutic outcomes.

Currently, three common mechanisms of cisplatin resistance (CR) in HNC cells have been reported [6,7,8,9]. The first is the nucleotide excision repair, wherein HNC cells survive cisplatin-induced DNA damage via the support of the DNA excision repair protein ERCC1 [10,11,12]. The second is the drug efflux; this can mediate cisplatin resistance by reducing intracellular drug levels. The ATP binding cassette transporter superfamily comprises drug efflux transporters encoded by multidrug resistance (MDR) genes. Cisplatin resistance has been shown to correlate with high expression of these MDR-related genes and leads to poor survival in HNC patients [13]. The third is resistance to cisplatin-induced apoptosis due to the suppression of apoptosis (IAP) proteins, such as c-IAP1, XIAP, and Apollon [14,15,16,17,18].

Although these mechanisms have been widely reported related to CR, they may not explain all the causes. To obtain a more comprehensive profile of the molecular mechanisms, in this study, we established isogenic sublines of HNC cells’ characteristics of CR. Bioinformatics software and data mining techniques were used to assess core molecular pathways and critical genes associated with CR. We identified a novel biological process (mitotic cell division) and demonstrated a hub gene (SPC25) that significantly contributed to cancer stemness and CR. Furthermore, a natural compound (celastrol) was found to exhibit a great cytotoxic effect on CR cells and suppress SPC25 expression. The results of this study may be further applied in personalized chemotherapy by sensitizing cisplatin to treat refractory HNC.

## 2. Results

### 2.1. Establishment of Cisplatin-Resistant (CR) Sublines of HNC Cells

**Figure 1:**
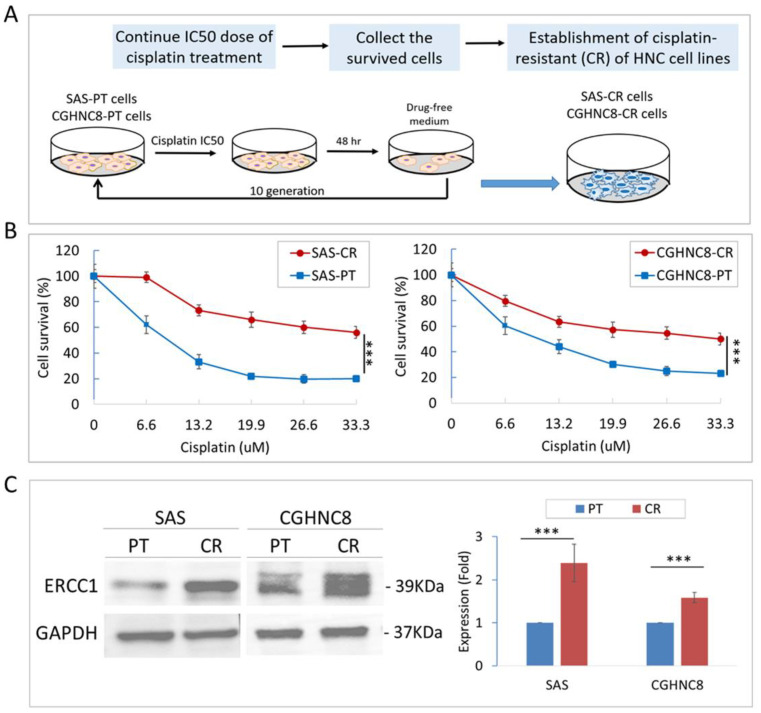
Establishment of cisplatin-resistant (CR) cells derived from head and neck cancer (HNC) cell lines. (**A**) The schematic presentation of the method to establish two CR sublines from two HNC cell lines, SAS and CGHNC8, via the chronic treatment of cisplatin (IC50 doses for 10 generations). (**B**) Viability of HNC parental (PT) cells and the respective CR cells were determined following cisplatin treatment. Cells were treated with various concentrations of cisplatin as indicated (0–33.3 uM) for 48 h, and cell viability was examined by MTS assay. The signal from untreated cells was considered to represent 100% viability. (**C**) The expression of the excision repair cross-complementing rodent repair deficiency, complementation group 1 (ERCC1) molecule was evaluated by immunoblot analysis and quantified by the determination of relative band density. The level of GAPDH expression was used as an internal control to determine relative protein expression. Statistical significance is indicated by *p*-values (***: *p* ≤ 0.001). Data are presented as the mean ± SD.

Two HNC cell lines (SAS and CGHNC8) were chronically exposed to cisplatin to establish cisplatin resistance sublines (SAS-CR and CGHNC8-CR). The process of the established protocol is summarized in Figure 1A. These CR sublines were subjected to various doses of cisplatin to validate their differential chemosensitivity. As shown in Figure 1B, the IC50s in SAS and CGHNC8 cells were 9.4 and 14.57 μM, respectively, while those in SAS-CR and CGHNC8-CR cells were greater than 33.3 μM. Compared to the parental cells, the survival rates of SAS-CR and CGHNC8-CR cells increased to approximately 2.78- and 2.15-fold, respectively, at a dose of 33.3 μM.

ERCC1 (excision repair cross-complementing rodent repair deficiency, complementation group 1), which has been widely reported to increase the proficiency of nucleotide excision repair and augment cisplatin resistance in tumor cells [19], was further examined to confirm CR property in these derived cell lines. As shown in Figure 1C, expression levels of ERCC1 were increased in both CR cell lines, with 2.4- and 1.6-fold elevation in the SAS-CR and CGHNC8-CR cells, respectively. The higher tolerance to cisplatin and the significant increase in ERCC1 biomarker protein demonstrated the successful establishment of these CR sublines.

### 2.2. The CR Sublines Exhibit Highly Invasive and Cancer Stemness Properties

**Figure 2:**
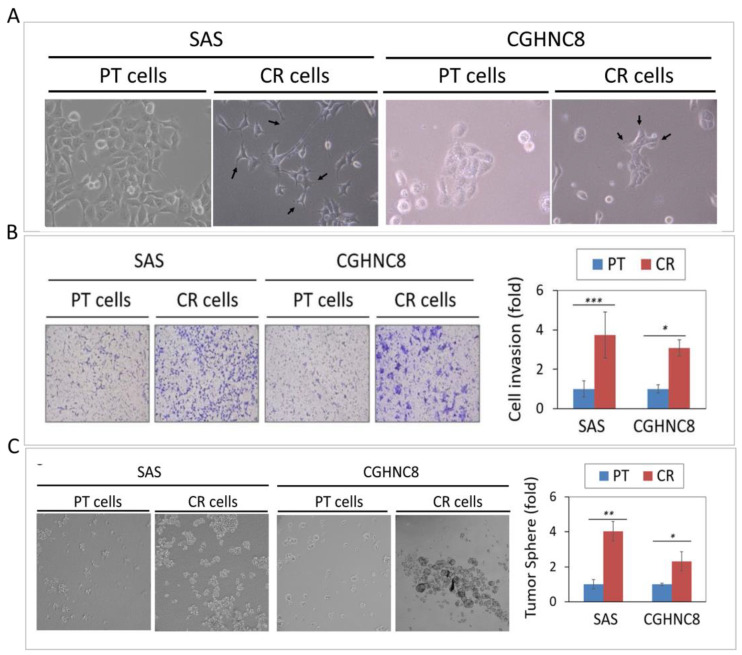

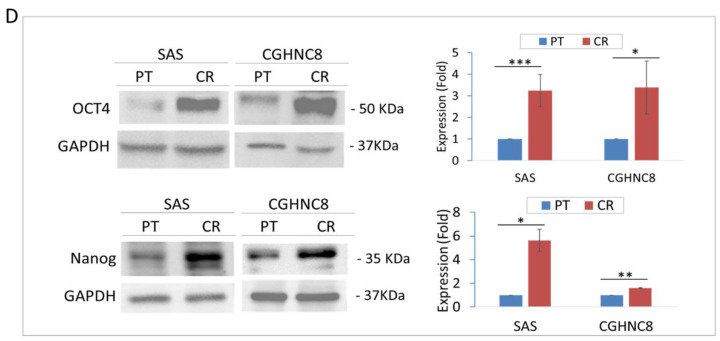
The CR sublines exhibit highly invasive and cancer stemness properties. (**A**) Morphological appearance of CR cells compared to the parental (PT) cells, as examined by microscopy. (**B**) Cell invasion of CR and PT cells, as determined by a Matrigel invasion assay. The numbers of cells that had invaded through the Matrigel to the bottom chamber were determined after 16 h. (**C**) Tumorsphere formation by CR and PT cells as examined by tumorsphere assay, as described in the Methods. The ability of tumorsphere formation was quantified by the determination of relative sphere numbers. (**D**) The expression of stemness regulators (Nanog and OCT4) were evaluated in SAS-CR and CGHNC8-CR cells by immunoblot analysis and quantified by the determination of relative band density. The expression level of GAPDH was used as an internal control. (*: *p* ≤ 0.05, **: *p* ≤ 0.01, ***: *p* ≤ 0.001).

In addition to increasing cisplatin resistance, we examined whether these CR cells may possess other malignant phenotypes. Because these CR cells showed blast-like morphology (Figure 2A), we speculated that these cells might alter their invasive ability. As determined by Matrigel invasion assay, the CR sublines showed higher cellular invasion, with 3.8- and 3.5-fold increases in SAS and CGHNC8 cells, respectively (Figure 2B). Recent studies have shown that cancer stem cells (CSCs) are responsible for tumor initiation, cancer metastasis, and resistance to conventional chemotherapy [20,21,22]. We, therefore, examined whether these CR sublines also possess cancer stemness property by using tumorsphere formation assay, a hallmark to assess the self-renewal capacity of CSCs. As shown in Figure 2C, the SAS-CR and CGHNC8-CR cells displayed higher tumorsphere formation, as evidenced by the larger size and increased number of the spheres. Consistently, the molecular expressions of the stemness regulators (Nanog and OCT4) were significantly increased in both SAS-CR and CGHNC8-CR cells (Figure 2D). These results indicated that the CR cells may co-exhibit with cancer stemness phenotype, which may be facilitated by pluripotent regulators.

### 2.3. Transcriptomic Profile and Functional Pathways Associated with CR in HNC

To obtain a comprehensive molecular profile associated with CR, the differential transcriptomes between HNC cell lines and the respective CR sublines were examined using Affymetrix GeneChip microarray analysis. After ANOVA analysis, a total of 647 genes exhibited differential expression genes (DEGs), with 345 upregulated and 302 downregulated in the CR sublines. Tables S1 and S2 list the top 100 upregulated and top 100 downregulated genes, along with the average fold changes in these two cell lines.

**Figure 3:**
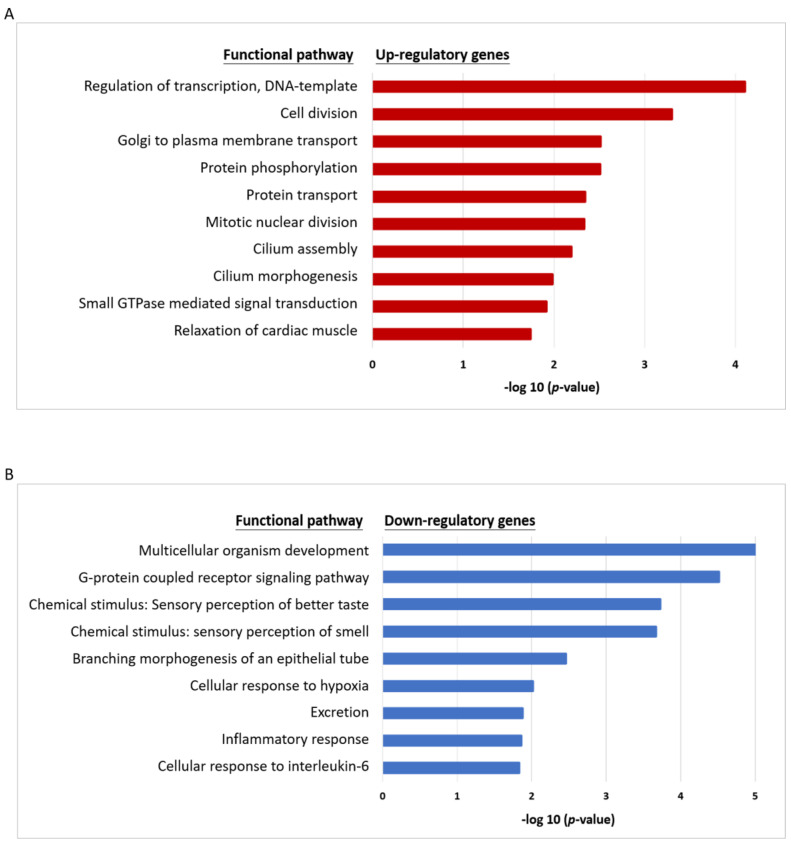
Bioinformatic analysis of the functional pathways associated with CR in HNC cells. (**A**) A list of the top 10 significant molecular functional pathways determined by the Database for Annotation, Visualization, and Integrated Discovery (DAVID) enrichment analysis of the 345 up-regulatory genes. (**B**) A list of the top 10 significant molecular functional pathways determined by DAVID enrichment analysis of the 305 down-regulatory genes. Bar chart representing the classification of Gene Ontology (GO) terms corresponding to biological processes (GOTERM_RP_ DIRECT). The enriched significance (*p* ≤ 0.05) values were negative base-10 log-transformed.

To determine whether this CR phenotype may relate to a certain biological process, the up- and downregulated DEGs were imported into the Database for Annotation, Visualization, and Integrated Discovery (DAVID) online software to analyze the associated functional pathways. Results of the top 10 up- and down-regulatory pathways are shown in Figure 3A,B. The enriched genes of each pathway are listed in Table S3. In the up-regulatory pathway, these CR molecules were found to be enriched most related to cell growth and comprised 3 of the 10 mechanisms: the transcriptional regulation associated with DNA-template, the regulation of cell division, and the mitotic nuclear division (Figure 3A). Other oncogenic pathways, such as small GTPase mediated signal transduction was also noted (Figure 3A). As for the down-regulatory pathway, these CR molecules were found to be enriched and significantly related to the chemical stimulus or stress response (Figure 3B). These included the regulatory mechanisms of sensory perceptions on bitter taste and smell, the response to hypoxia, and interleukin-6 mediated inflammation. All these results indicate that the induction of the CR phenotype may also involve wide ranges of cellular and molecular alterations to maintain its homeostasis in HNC cells.

### 2.4. Panel Molecules that Were Upregulated in CR Cells and Led to Poor Prognosis in HNC Patients

**Figure 4:**
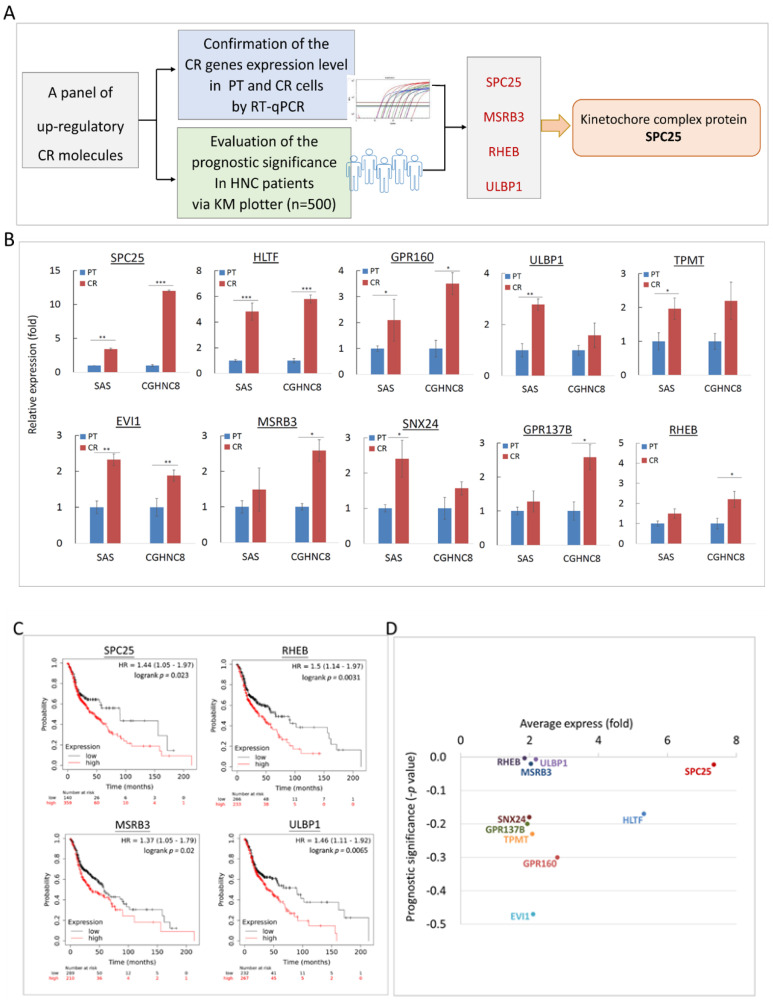
Prognostic significance of CR associated genes in HNC patients. (**A**) Flow diagram representing the enrichment process on HNC patients for survival analysis by KM-Plotter. (**B**) Relative expressions of 10 CR genes between parental (PT) cells and CR sublines of the SAS and CGHNC8 cell lines using the RT-qPCR method. The expression levels are shown by bars. (**C**) Prognostic significance determined by the Kaplan–Meier Plotter online tool using the head–neck squamous cell carcinoma dataset (*n* = 500). (**D**) The overall view of 10 CR gene expressions (*x*-axis) with the prognostic significance (*y*-axis) for each gene. Values toward the top are signified higher levels of overexpression in CR cells, and those further to the right represent more clinical significance with poor prognosis (shown by the subtractive *p*-value) in HNC patients (*: *p* ≤ 0.05, **: *p* ≤ 0.01, ***: *p* ≤ 0.001).

To determine the potential clinical association of these CR genes in HNC patients, a panel of 10 up-regulatory molecules were selected and subjected to gene expression analysis and prognostic evaluation. The process of this validation protocol is summarized in Figure 4A. For gene expression analysis, RT-qPCR assays were performed to examine the levels of these panel molecules in HNC parental cells and the CR sublines. As shown in Figure 4B, all 10 molecules were significantly elevated in the CR sublines. These results confirmed our microarray analysis and implied the trustworthy value of the CR transcriptomic study.

For the clinical association study, the panel of 10 up-regulatory CR molecules was uploaded to the KM-Plotter suite for survival analysis. The KM-Plotter is a web-based tool to evaluate the association of multigene expression levels with patient survival in various cancers [23,24]. In this study, the HNC cohort (*n* = 500) was used. The most significant results are shown in Figure 4C. As shown, overexpression of these molecules was highly correlated to poor survival in HNC patients. These were SPC25 (*p* = 0.023), MSRB3 (*p* = 0.020), RHEB (*p* = 0.003), and ULBP1 (*p* = 0.006). To parallelly assess the significance of gene upregulation and clinical prognostic values, Figure 4D was plotted to show these two parameters for each CR molecule. As shown, several molecules were distinguished, such as SPC25, MSRB3, RHEB, and ULBP1. These results represented a panel molecule that modulates CR and leads to poor prognosis in HNC.

### 2.5. Cmap Analysis Identified Celastrol as a Potent Compound to Overcome CR

**Figure 5:**
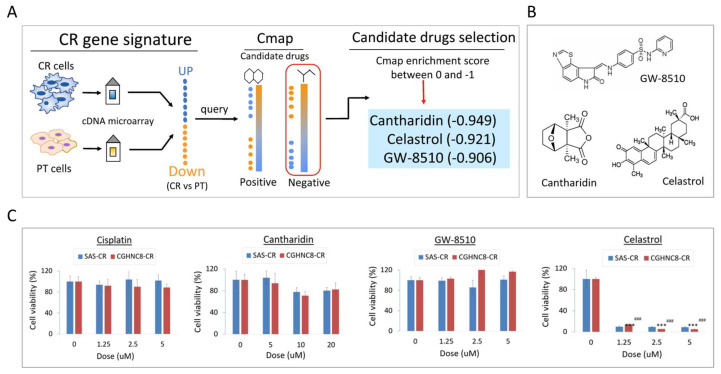

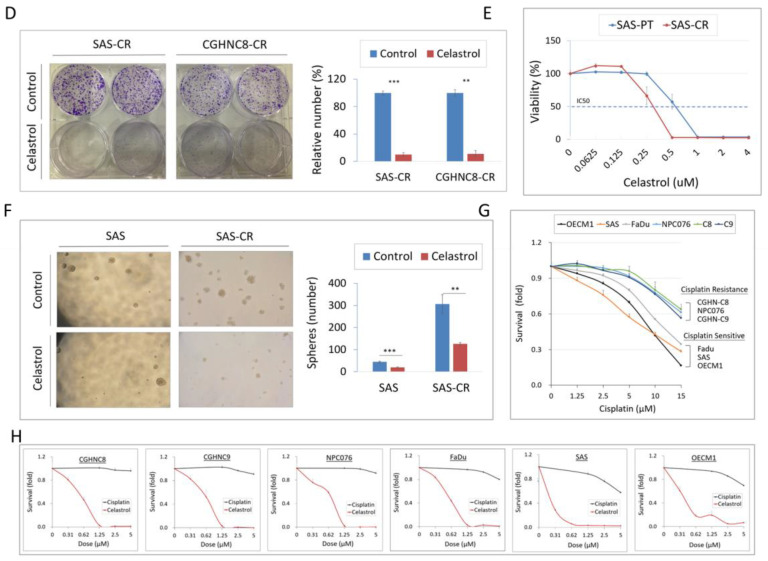
Connective Map (Cmap) analysis identified celastrol as a potent compound to overcome CR. (**A**) The schematic illustration of the process for exploring potential compounds to reverse CR. The CR gene signatures were used to query the Cmap database, and the compounds ranked by the enrichment score <0 were selected. (**B**) Chemical structure of the top three negative correlated compounds queried by Cmap analysis, as cantharidin, celastrol, and GW-8510. (**C**) Assessment for the drug efficacy on the CR cells. The SAS-CR and CGHNC8-CR cells were exposed to cisplatin, GW-8510, cantharidin, or celastrol at various doses for 48 h, and the cell viability was determined by MTS assay. (**D**) The efficacy of celastrol on the growth suppression of CR cells, as determined by using colony formation assay. SAS-CR and CGHNC8-CR cells were treated with 0.5 μM celastrol, following incubation for 7 days to allow colony formation. Cell colonies were stained with 5% crystal violet. (**E**) The differential effect of cytotoxicity by celastrol for SAS parental and SAS-CR cells. The dose–response curves with IC50 values for celastrol were established on SAS-PT and SAS-CR cells after treatment with various doses of celastrol for 2 days. (**F**) The differential effect of tumorsphere formation by celastrol for SAS parental and SAS-CR cells. Cells were grown in tumorsphere culture medium on ultralow attachment plates. After 14 days, tumorspheres were enumerated using microscopy. (**G**,**H**) The cytotoxic effect of cisplatin and celastrol in a panel of HNC cell lines. CGHCN8, CGHNC9, NPC076, Fadu, SAS, and OECM1 were treated with cisplatin for 48 h, and cell viability was performed by MTS assay. *p*-values indicate statistical significance (** *p* ≤ 0.01, *** *p* ≤ 0.001).

To explore a novel drug that may overcome CR in HNC cells, the Connective Map (Cmap) analytic strategy was employed to explore potential compounds. The conception of applying this strategy is shown in Figure 5A. After uploading the upregulatory genes from CR transcriptomic profile and querying for the Cmap database with the connectivity score <0, the top 3 negative correlation compounds were filtered out, as cantharidin (−0.949), celastrol (−0.921), and GW-8510 (−0.906). The chemical structures of these compounds are shown in Figure 5B.

To determine whether these compounds may introduce higher cytotoxicity compared with cisplatin, the cytotoxic analyses were performed in two CR sublines (SAS-CR, CGHNC8-CR). Results are shown in Figure 5C. There were no cytotoxic effects in these CR cells at 5 μM of either cisplatin, cantharidin, or GW-8510. However, strong cytotoxicity was found by celastrol, even at a dose lower than 1.25 μM. This cytotoxic effect of celastrol was further confirmed by colony formation in CR cells. As shown in Figure 5D, these CR cells were extremely sensitive to celastrol, with less than 10% of cell colony were found when treated with 0.5 μM of this compound. These results suggest that celastrol rendered a marvelous effect to overcome CR in HNC cells.

To assess whether celastrol may have differential cytotoxicity on cisplatin-respective cells and CR cells, the survival analysis was performed parallel in SAS parental cell line and its CR subline. As shown in Figure 5E, the cytotoxic effects of celastrol were obviously found in both cell lines. However, the CR subline exhibited stronger cytotoxicity in response to this compound, with a 40% reduction in the IC50 dose compared to the parental cells (0.52 and 0.31 μM, respectively, in parental and CR cells). Consistently, although CR cells augmented tumorsphere formation, celastrol was effective on this aggressive phenotype and suppressed tumorspheres significantly (Figure 5F). Furthermore, the cytotoxic effects of celastrol were validated in three intrinsic CR cell lines (CGHNC8, CGHNC9, NPC076) and effective to intrinsic cisplatin-sensitive cells as well (SAS, OECM1, Fadu) (Figure 5G,H). Taken together, our results suggest that celastrol may be a potent compound to overcome cisplatin resistance in HNC cells.

### 2.6. Silencing Kinetochore Protein SPC25 Increased Cisplatin Sensitivity in HNC Cells

**Figure 6:**
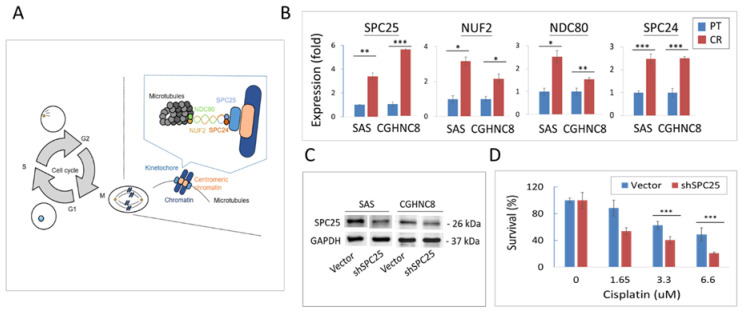

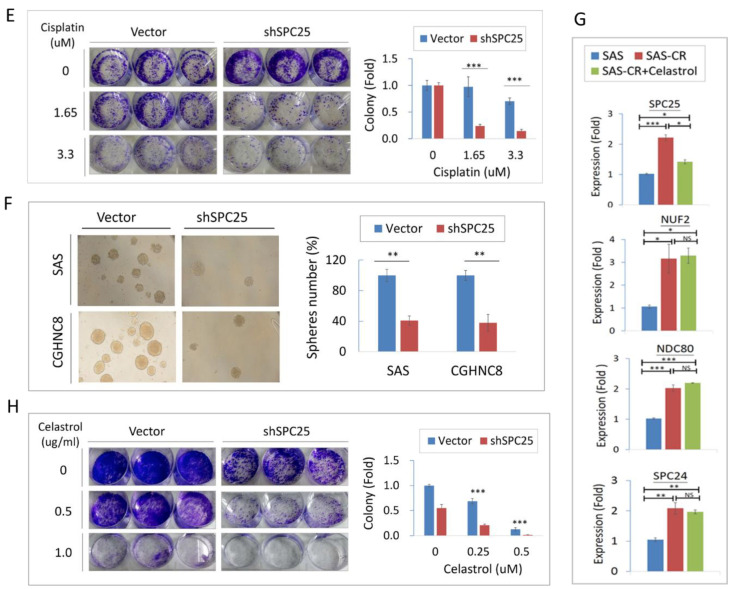
Silencing kinetochore protein spindle pole body component 25 (SPC25) contributes to cisplatin resistance. (**A**) Schematic diagram of the microtubule–kinetochore interface and NDC80 kinetochore complex proteins. (**B**) The expression level of NDC80 complex proteins (NDC80, NUF2, SPC24, and SCP25) in parental and CR cells as examined by RT-qPCR. (**C**) Reduction in SPC25 expression in SPC25-specific silencing cells. After transfection of SPC25-specific shRNA plasmids or the vectors, the stable transfected cells were selected by using G418. The efficiency of SPC25 silencing was verified by the protein expression as determined by Western blotting. The level of GAPDH expression was used as an internal control to determine relative protein expression. The effect of SPC25 silencing (shSPC25) on the cisplatin sensitivity as determined using survival, as determined by (**D**) MTS survival, or (**E**) colony formation assays in SAS cells. (**F**) The effect of SPC25 silencing (shSPC25) on cancer stemness as determined by tumorsphere assay in SAS and CGHNC8 cells. (**G**) The specific effect of celastrol on the inhibition of SPC25 expression in SAS-CR cells. The SAS cells were treated with 0.5 μM c celastrol, and the gene expression level was performed by RT-qPCR. (**H**) Effect of growth suppression by celastrol and SPC25-silencing. The shSPC25 or the vector-transfected SAS cells were treated with various doses of celastrol, and cell colony formation was determined after 7 days. *p*-values indicate statistical significance (* *p* ≤ 0.05, ** *p* ≤ 0.01, *** *p* ≤ 0.001).

In our defined panel molecules that were upregulated in CR cells and led to poor prognosis in HNC patients, SPC25 showed at the domineering place (Figure 4D). We, therefore, investigated how this molecule may contribute to CR in HNC. SPC25 (spindle pole body component 25), a component of the central kinetochore NDC80 protein complex, plays an essential role in mitotic chromosome segregation [25,26,27,28]. This kinetochore protein complex consists of several molecular components, including SPC25, NUF2, NDC80, and SPC24, which regulates microtubule attachment and spindle assembly during mitosis (Figure 6A). To examine whether this kinetochore complex may participate in a role in CR, the differential expressions of these molecules were determined in HNC parental cells and the CR sublines. As shown in Figure 6B, all the complex proteins were elevated expressions in both SAS- and CGHNC8-CR sublines, with the levels of SPC25 (3.8- and 5.6-fold), NUF2 (3.2- and 2.2-fold), NDC80 (2.5- and 1.5-fold), and SPC24 (2.4- and 2.5-fold). These results indicated that kinetochore complex proteins play important roles in the regulation of CR phenotype.

The specific function of SPC25 in cisplatin sensitivity was further assessed by performing shRNA knockdown experiments via stably SPC25-depleted cell lines (SAS-shSPC25 and CGHNC8-shSPC25). After the successful knockdown, SPC25 expression in HNC cells (Figure 6C), the effects of cisplatin sensitivity, and tumorsphere formation were evaluated. As shown in Figure 6D, cell survival was reduced with increasing doses of cisplatin in two cell lines, while the shSPC25 cells exhibited significant decreasing levels compared to the control cells. At the 2 μg/mL of cisplatin treatment, cell survival reduced to 22% in the shSPC25 cells compared to 51% in the control cells. Consistently, in response to cisplatin treatment, the shSPC25 cells showed a significant reduction in colony formation compared to the control cells (Figure 6E). At 1 μg/mL of cisplatin treatment, the cell colony reduced to 9% in shSPC25 cells compared to 68% in the controls. For tumorsphere formation, shSPC25 cells also showed substantially fewer numbers of tumorspheres compared to the controls, by a decrease to approximately 40% in SPC25 silencing cell lines compared to the controls (Figure 6F). All these results suggest that SPC25 was a critical molecule contributing to CR, while knockdown of this molecule sensitized the cytotoxic effect of cisplatin in HNC cells.

We further examined whether celastrol, the defined specific compound to overcome CR, may affect kinetochore complex proteins. RT-qPCR analyses were performed to determine the differential expressions of these proteins (SPC25, NUF2, NDC80, and SPC24) in SAS parental cells, SAS-CR cells, and SAS-CR cells treated with celastrol. Results are shown in Figure 6G. Although all proteins were elevated in CR cells compared to parental cells, only SPC25 showed responsiveness to celastrol. This result indicates that the cytotoxicity induced by celastrol in CR cells may be via targeting the SPC25 molecule. Finally, the combinational effect of celastrol and SPC25-silencing on cell growth was assessed using colony formation assay. As shown in Figure 6H, either celastrol or SPC25-silencing inhibited cell colony formation. However, this combined treatment led to a synergistic effect, with 72% of colony formation when treated by celastrol (0.25 μM) in vector cells while 21% of the colony in SPC25-silencing cells (*p* < 0.01). Similar results were also found in the cells treated with the higher dose of celastrol (0.5 uM). These results suggest that SPC25-silencing sensitized the cytotoxicity of celastrol in HNC cells.

## 3. Discussion

The efficiency of cisplatin is limited by drug resistance in HNC patients. To overcome CR, we employed a systemic strategy to comprehensively examine possible molecular mechanisms and explore potent compounds as therapeutic targets. A few points may highlight our works presented in this study. (1) The transcriptomic profile and molecular mechanisms related CR were established. The mitotic division, a novel mechanism, was revealed to contribute to CR significantly. (2) A panel of molecular signatures associated with CR was identified as SPC25, MSRB3, RHEB, and ULBP1. These molecules were overexpressed in CR cells and correlated with poor survival in HNC patients. (3) A kinetochore protein SPC25 was demonstrated to play an imperative role in CR. Silencing this molecule suppressed cancer stemness and attenuated CR in HNC cells. (4) Celastrol was demonstrated as an effective compound to inhibit SPC25 expression and induce substantial cytotoxicity in CR cells. Celastrol may be used as a novel drug to overcome CR for the treatment of refractory HNC.

In this study, two isogenic CR subline cells of HNC were established. The integrated analysis of DEGs revealed that the mitotic division was a novel functional mechanism related to CR (Figure 3). In this mechanism, several molecules were identified with altered expressions in CR cells, including those which regulate mitotic spindle organization (MAPRE3, SPDL1, KATNA1, TUBA1A) and modulate chromosome condensation (NUF2, SPC25, and SKA1) (Table S3) [25,26,27]. The kinetochore complex, originally reported to be responsible for mediating the interaction of sister chromatids with mitotic spindle during mitosis [25], was found to play a critical role in CR in this study. This kinetochore complex consists of three parts: (i) centromeric heterochromatin, (ii) the internal plate, and (iii) the outer plate. The outer plate structure contains the NDC80 complex, including proteins NDC80, NUF2, SPC24, and SPC25 [26,27,28]. In this study, we first noted that several kinetochore complex proteins (SPC25, NDC80, NUF2, and SPC24) were significantly overexpressed in CR cells (Figure 6B). These results provide the insight that the kinetochore proteins may play roles in overcoming cisplatin-induced DNA damage, leading to sustained genetic instability in CR cells.

In this study, a panel of molecules was confirmed to upregulated the CR cells (Figure 4B). Our findings are supported by other investigators, showing that these molecules also possess oncogenic functions with several facets. For example, GRP160, a G-protein-coupled receptor, was found to promote tumor growth by inhibiting apoptosis [29]. EVI1 (the transcription factor ecotropic viral integration site 1) was overexpressed in patients with myeloid malignancy and associated with poor prognosis [30,31]. HLTF (the transcription factor helicase-like transcription factor) was reported to modulate genomic stability by regulating chromatin remodeling and the DNA repair mechanism [32,33]. For those molecules that were demonstrated to be correlated with poor prognosis in HNC patients (Figure 4C), RHEB was also found overexpressed in prostate cancer, associated with poor prognosis [34], and promoted cell growth via the regulation of the mTOR signaling pathway [35,36]. Elevated expression of MSRB3 (methionine sulfoxide reductase), a reactive oxygen species scavenging enzyme, was shown to be associated with poorer survival in gastric cancer [37,38]. The ULBP1 (UL16 binding protein 1), known as a “stress-induced ligand”, was upregulated in cancer cells and related to the function of escaping immune surveillance [39]. Taken together, our defined CR related molecules exhibit a novel biomarker panel, which may be further used to assess drug sensitivity for prediction and prognostic application.

The potential effect of the kinetochore integrity on chemo-drug sensitivity has not been addressed previously. In the outer plate of kinetochore complex proteins, SPC25 exhibited a prominent position related to CR in terms of high expression level and clinical prognostic relevance ( and Figure 4DFigure 6B). SPC25 mediates chromosome alignment and spindle formation essential for chromosome segregation to avoid DNA break [40,41,42,43,44]. Recently, SPC25 has been found to participate in numerous malignant phenotypes, including proliferation, survival, stemness, and metastasis in various cancers [43,44,45,46,47,48,49,50]. These effects may dysregulate several oncogenic signaling pathways, such as Wnt signaling, TGF-β signaling, and the SUMOylation pathway [45,46,47,48,50]. Our present study is consistent with these findings. We demonstrate that SPC25 was upregulated in cisplatin-resistant cells and that silencing of this molecule increased cisplatin sensitivity to HNC cells (Figure 6). To our best knowledge, this is the first report of the direct link between SPC25 level and cisplatin resistance. We also noted that the overexpression of SPC25 correlated with poor survival in HNC (Figure 4D). This clinical relevance of prognostic association was similarly found in other cancer types (lung, breast, and prostate) [45,47,48]. Therefore, SPC25 may be used as a predictive biomarker, which can be further applied in personalized chemotherapy or as a sensitizing target for cisplatin treatment in HNC.

The Cmap strategy has been recently used in drug discovery to reverse a biological effect based on gene expression profile and drug-related signature. By this approach, celastrol has been identified as an effective drug to reverse CR phenotype in HNC cells (Figure 5C,D). Celastrol is a chemical compound isolated from the root extracts of *Tripterygium wilfordii* (Thunder god vine) and *Celastrus regelii* [51]. Recently, this compound has been noted and reported possessing anti-cancerous activity. This anticancer effect may involve several mechanisms, including the inhibition of cell growth [52], suppression of cell invasion or angiogenesis [53,54], or the induction of apoptosis [55,56,57,58]. At the molecular level, these functions may be achieved by the suppression of NF-κB activity [56,59], inhibition of PI3/Akt signaling pathway [57], or induction of the heat shop protein (Hsp90) mediated cell death [55,60,61,62].

In this study, we found that celastrol may sensitize cisplatin and lead to a potent cytotoxic effect in CR cells (Figure 5C,D). Our results were in agreement with other investigators, which showed the synergistic effects of combined use of celastrol with other anticancer drugs. These were the combined use of celastrol with lapatinib in hepato-carcinoma cells [63], with SAHA [59], or gefitinib in lung cancer cells [55], and with doxorubicin in colon cancer cells [64]. Furthermore, we found that celastrol also actively suppressed tumorsphere formation (Figure 5F), indicating it may be effective for stemness-like cancers. The marvelous effect of celastrol may involve the specific inhibition of SPC25 (Figure 6G), an essential molecule of kinetochore protein during mitotic division. We further noted that the combination treatment of celastrol and SPC25-silencing significantly augment the anticancer efficacy by the suppression of cell colony formation (Figure 6H). The synergistic cytotoxicity of celastrol and SPC25-silencing may due to their reciprocal sensitization, resulting in the catastrophe in kinetochore structure, growth termination, and cell killing. Thus, this combination regimen of celastrol and SPC25-silencing may be further developed for the treatment of refractory cancers.

## 4. Materials and Methods

### 4.1. Establishment of Cisplatin-Resistant (CR) Sublines of HNC

The oral cancer cell lines SAS and CGHNC8 were used in this study [65,66]. The cells were maintained in DMEM medium (Gibco, ThermoFisher Scientific, Inc., MA, USA), with 10% fetal bovine serum and 1% antibiotics. All cells were cultured at 37 °C in a humidified atmosphere, with 5% CO_2_. Cis-Diamineplatinum (II) dichloride (cisplatin) (Pharmachemic, Utrecht, the Netherlands) was used to generate cisplatin-resistant (CR) sublines. When cell confluence reached 70%, cultures were treated with cisplatin at IC50 (50% inhibitory concentration) for 48 h. The medium was changed to a drug-free medium until cells reached 90% confluence. The first-generation subline was obtained from the surviving fraction of the parental cells treated with the respective IC50 doses. Similarly, the second-generation subline was obtained from the surviving fraction of first-generation cells treated with the respective IC50 doses. Finally, ten generations of CR sublines were established, which were designated as SAS-CR and CGHNC8-CR cell lines.

### 4.2. Transcriptional Profiling and Validation of CR Genes in HNC

To globally determine gene expression profiles associated with CR in HNC cells globally, cDNA microarray analysis was used to compare differentially expressed genes (DEGs) between HNC parental cell lines and their CR sublines. The RNA extraction and microarray analyses were performed, as previously described [67,68]. Briefly, the total RNA was extracted using the TRIzol Reagent (Gibco BRL). The complementary RNA was hybridized to the Human Affymetrix Exon 1.0ST microarray gene chip and scanned using an Affymetrix GeneArray 2500 scanner (Affymetrix, Santa Clara, CA, USA). Raw signal intensities were imported into the Partek Genomics Suite (Partek, St. Louis, MO, USA) and were normalized using quantile normalization. The significance of differentially expressed genes was determined using the ANOVA analytical method with the cut-off criteria |fold change| > 1.5 and *p*-value < 0.05 between parental cells and CR sublines.

Differentially expressed genes between HNC cell lines and CR sublines were evaluated using the RT-qPCR method as previously described [21]. Briefly, the cDNA synthesis and qPCR were performed using the MiniOpticon^TM^ real-time PCR detection system and SYBR Green Supermix reagents. The primers used in this study, including those for KITLG, SPC25, KCNJ2, HLTF, RAB23, MSRB3, ULBP1, RHEB, STAB1, TPMT, GPR160, EVI1, SPC24, NDC80, and NUF2, are listed in Table S4. The expression of GAPDH was used as a reference.

### 4.3. Analysis of Functional Pathways and Prognostic Significance in HNC

To determine the biological pathways associated with cisplatin resistance, the DEGs identified in the microarrays were analyzed using computational methods. Gene-annotation enrichment analysis was performed using the web-based gene enrichment analysis tool, the Database for Annotation, Visualization, and Integrated Discovery (DAVID) Bioinformatics Resources 6.8 (https://david.ncifcrf.gov/) [69]. Significantly enriched Gene Ontology (GO) terms for DEGs were explored and grouped. Significantly enriched functional terms (adjusted *p*-values < 0.05) for up- or downregulated genes were reported.

The KM-Plotter online tool (http://kmplot.com/analysis) was also used to evaluate the prognostic significance of CR-associated genes in HNC patients, as previously described [70]. The HNC cohort dataset (*n* = 500) was analyzed. High- and low-risk groups were classified using an optimization algorithm in the order of the prognostic index according to each gene expression level. A Kaplan–Meier analysis was performed to evaluate overall survival. The hazard ratios (HRs) with 95% confidence intervals (CIs) were calculated using the log-rank test.

### 4.4. Cellular Protein Extraction and Immunoblot Analysis

Cellular protein extraction and immunoblot analysis were performed as previously described [71,72]. Briefly, cellular proteins were extracted using a detergent containing CHAPS lysis buffer, separated by SDS-polyacrylamide gel electrophoresis, and transferred to a nitrocellulose membrane. The membrane was hybridized to primary antibodies: anti-ERCC1 (D61F5, Cell Signaling, Danvers, MA, USA), anti-OCT4 (11263-1-AP, Proteintech, Rosemont, IL, USA), anti-Nanog (EPR2027(2), Abcam, Cambridge, MA, USA), anti-SPC25 (ab20679, Abcam, Cambridge, MA, USA), and anti-GAPDH (AF7021, Affinity Biosciences, Cincinnati, OH, USA). The membrane was subsequently incubated with a secondary antibody conjugated with horseradish peroxidase (Santa Cruz Biotech, CA, USA). The membranes were developed using an ECL developing solution (Merck Millipore, MA, USA) followed by autoradiography. All immunoblot experiments were performed at least two times. The original blots are shown in the Figure S1, and one typical result is presented. The protein expression shown in each band was quantified after normalization to the GAPDH expression level. The error bars shown in the relevant figures indicated the standard deviation of the quantification results.

### 4.5. Plasmid Construction and Cellular Transfection

Construction of the short heparin (sh)-SPC25 plasmid and the following transfection experiments were performed as previously described [71]. Sense and antisense hairpin nucleotides complementary to SPC25 mRNA were also generated. The sequence 5’- CTT AAG GAA TAT TCT AGG AAG CTT GCT AGA ATA TTC CTT AAG -3’ was cloned into the pTOPO-U6 plasmid or pCI-neo vector. Plasmids were transfected into cells using Lipofectamine 2000 reagent and OPTI-MEM medium (Invitrogen, Waltham, MA, USA) according to the manufacturer’s instructions. Cellular clones that were stably transfected with shSPC25 plasmid were selected using the neomycin antibiotic G418. The clones of shSPC25-2 in SAS and shSPC25-3 in CGHNC8 were selected, which were confirmed the silencing effect by immunoblot blot assay. (Figure S1).

### 4.6. Cell Survival, Colony Formation, and Chemosensitivity Analyses

The cytotoxic effect of cisplatin was determined using survival and colony formation assays. For viability assays, 5 × 10^3^ cells were seeded onto 96-well plates and were later incubated for 16 h. Various doses of cisplatin were added. After incubating for 48 h, cell viability was measured using the MTS assay according to the manufacturer’s instructions (Promega, Madison, WI, USA). Relative cell viability was calculated as the percentage of viable cells treated with cisplatin compared to untreated control cells. The colony formation assay was performed as previously described [71]. Cells were treated with cisplatin, as indicated. Cells were continuously cultured for 7 to 10 days. Colonies were stained with crystal violet and were photographed.

### 4.7. Cell Invasion and Tumorsphere Assays

Cell invasion and tumorsphere assays were performed as previously described [72,73]. For invasion assay, the cells in DMEM with 1% FBS were seeded into Transwell invasion chambers (BD Biosciences) coated with Matrigel (Merck). The lower chambers contained 10% FBS to trap cells. After 16 h, the cells invading the reverse side of the membrane were fixed, stained, and photographed. For tumorsphere assay, the cells were seeded on ultra-low attachment plates (Merck, Darmstadt, Germany) in the DMEM/F-12 serum-free medium (Gibco, Waltham, MA, USA) with B27 supplement (Invitrogen) and growth factors bFGF and EGF. The plates were incubated at 37 °C for 10 days. Cell spheres were visualized and enumerated using light microscopy.

### 4.8. Connectivity Map (Cmap) Analysis to Search Potential CR Sensitizers

The Connectivity Map (Cmap) database (http://www.broadinstitute.org/cmap/) was used to search for the potential compounds to overcome cisplatin resistance globally. This Cmap system contains a pool of compound signature database, which includes 6100 drug-response microarrays on 1309 drug compounds affecting various cancer cell lines [74]. This database has been recently used in novel drug discovery by integrative analysis of a gene expression profile and drug-related signature [75,76]. In this study, the top 100 DEGs from the transcriptomic profile of CR cells were uploaded and queried with Cmap database to build up positive and negative correlations with drug compounds. The output result showed a connectivity score, which ranges from 1 to −1. The data of positive enrichment score indicates the same trend of gene expression profile between CR and the corresponding perturbagen, so that the queried compounds may function as potential inducers of CR. On the contrary, the data of negative enrichment score indicates the opposite trend of gene expression profile between CR and the corresponding perturbagen, so that the queried compounds may serve as potential sensitizing drugs to reverse CR. After querying with the Cmap database, the candidate CR compounds were selected with the criteria of negative enrichment score.

### 4.9. Statistical Analysis

For comparisons between the means of two variables, a two-tailed unpaired Student’s *t*-test was used. For comparisons among multiple variables, one-way ANOVA was used. All statistical analyses were conducted using a significance level of α = 0.05 (*p* < 0.05).

## 5. Conclusions

Cisplatin is the first-line chemotherapy agent for HNC, but its therapeutic efficiency may be hampered by cisplatin resistance (CR). In this study, we have employed a systemic strategy aimed at overcoming CR. We have established a CR related transcriptomic profile, determined the functional mechanisms (such as mitotic division), identified a prognostic signature, and explore potent compounds to reverse CR. A hub molecule SPC25 was defined as playing an imperative role contributing to CR. Silencing this molecule suppressed cancer stemness and attenuated CR in HNC cells. Celastrol, a natural extract compound, was demonstrated as a SPC25 inhibitor and possessed high cytotoxic efficacy in CR cells. The development of SPC25 inhibitors, such as the application of celastrol, maybe a novel strategy to sensitize cisplatin for the treatment of refractory HNC.

## Supplementary Materials

The following are available online at https://www.mdpi.com/2072-6694/12/11/3482/s1, Table S1: Information of top 100 genes upregulation in the CR cells. The average fold change and *p*-value were listed, Table S2: Information of top 100 genes upregulation in the CR cells. The average fold change and *p*-value are listed, Table S3: Gene ontology analysis of all CR-associated genes, Table S4: List of primers used in the present study. Figure S1: Original immunoblotting images presented in the paper.
