# Supplementary material for: A Combined Systemic Strategy for Overcoming Cisplatin Resistance in Head and Neck Cancer: From Target Identification to Drug Discovery
Source: Cancers (Basel). 2020 Nov 23;12(11):3482. doi: 10.3390/cancers12113482 (PMC7700594; doi:10.3390/cancers12113482)
Supplement: Supplementary file 1 [file cancers-12-03482-s001.pdf]

Supplementary Materials

A combined systemic strategy for overcoming cisplatin resistance in head and neck cancer: from target identification to drug discovery

Yin-Ju Chen, Guo-Rung You, Meng-Yu Lai, Long-Sheng Lu, Chang-Yu Chen, Lai-Lei Ting, Hsin-Lun Lee, Yazuka Kanno, Jeng-Fong Chiou and Ann-Joy Cheng

Immunoblot show in Figure 1C.

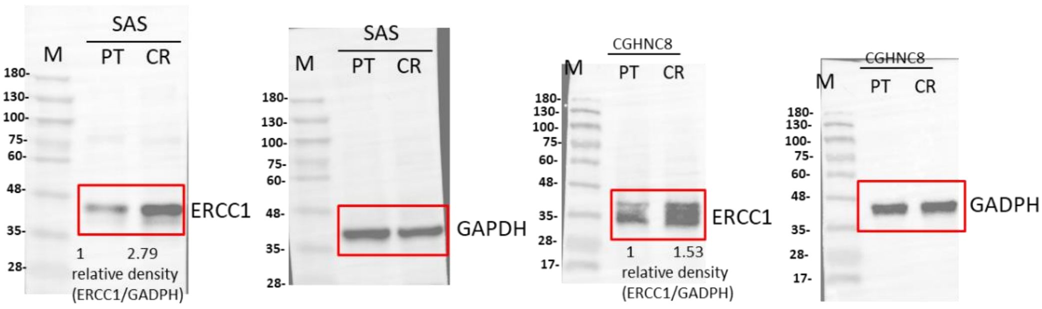

Immunoblot show in Figure 2.

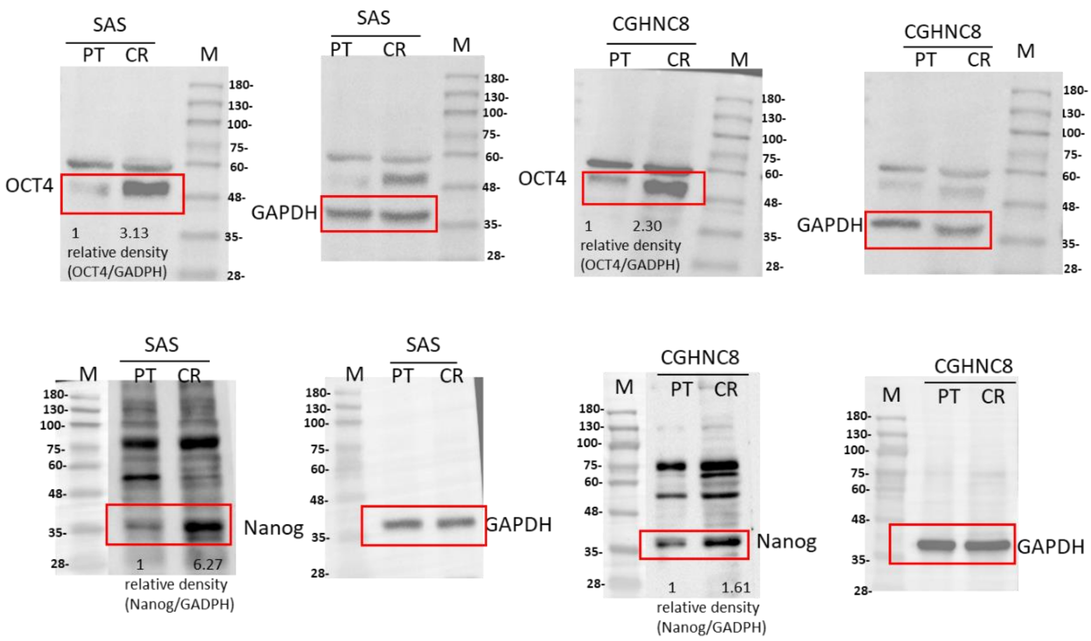

Immunoblot show in Figure 6.

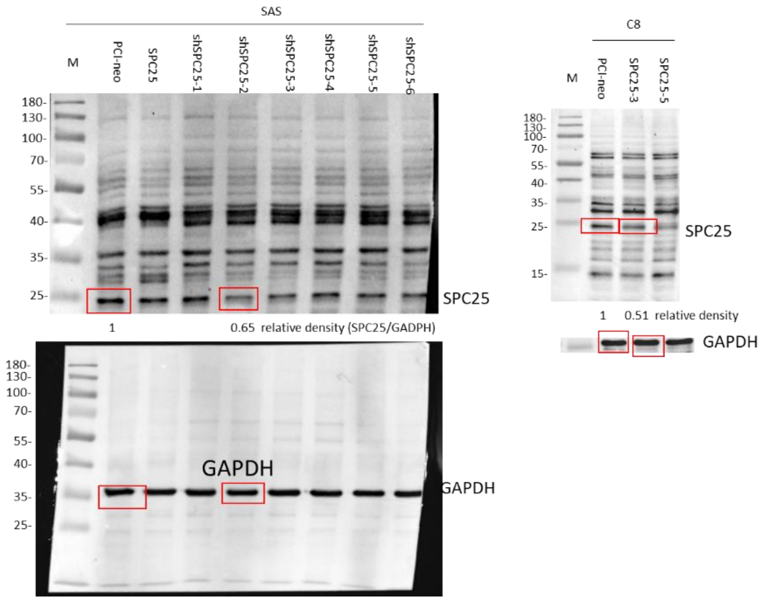

Figure 1. Original immunoblotting images presented in the paper.

Table 1. Information of top 100 genes up-regulation in the CR cells. The average fold change and *p*-value were listed.

Gene Symbol	Gene Name	Fold change	*p*-Value
HIGD2A	HIG1 domain family, member 2A	4.57	6.29E-04
TUBA1B	Tubulin, alpha 1b	3.98	1.41E-02
SLC25A33	Solute carrier family 25, member 33	3.62	4.43E-02
PROS1	Protein S (alpha)	3.25	2.29E-02
ULBP1	UL16 binding protein 1	3.17	4.83E-02
ETV1	Ets variant 1	3.15	6.45E-03
KITLG	KIT ligand	3.03	1.54E-02
RAP1B	RAP1B, member of RAS oncogene family	3.00	2.01E-02
LAMA4	Laminin, alpha 4	3.00	2.75E-03
CCDC23	Coiled-coil domain containing 23	2.84	3.40E-02
ZNF417	Zinc finger protein 417	2.62	1.22E-02
TPMT	Thiopurine S-methyltransferase	2.34	1.38E-02
RWDD2A	RWD domain containing 2A	2.32	1.80E-02
TMEFF1	Transmembrane protein with EGF-like and two	2.32	2.99E-02
ZNF675	Zinc finger protein 675	2.31	9.09E-05
C7orf58	Chromosome 7 open reading frame 58	2.26	2.39E-03
UTRN	Utrophin	2.19	1.28E-02
MRPS18C	Mitochondrial ribosomal protein S18C	2.19	2.55E-02
MSRB3	Methionine sulfoxide reductase B3	2.15	3.19E-02
KIAA1841	KIAA1841	2.14	1.67E-02
STX11	Syntaxin 11	2.14	1.46E-02
NEXN	Nexilin (F actin binding protein)	2.13	3.79E-02
TTLL7	Tubulin tyrosine ligase-like family, member 7	2.11	4.82E-02
NDUFA	NADH dehydrogenase (ubiquinone) 1 alpha	2.11	1.03E-02
MOP-1	MOP-1	2.11	4.04E-03
SPC25	SPC25, NDC80 kinetochore complex component,	2.10	1.48E-02
LSM5	LSM5 homolog, U6 small nuclear RNA	2.09	4.75E-02
DST	Dystonin	2.07	1.57E-03
KRTAP4-	Keratin associated protein 4-12	2.06	1.81E-05
EVI1	Ecotropic viral integration site 1	2.06	4.58E-02
SERPINI	Serpin peptidase inhibitor, clade I (neuroserpin),	2.06	5.56E-03
HIVEP	Human immunodeficiency virus type I enhancer	2.06	1.47E-02

C6orf19	Chromosome 6 open reading frame 199	2.04	1.67E-02
PHLDB	Pleckstrin homology-like domain, family B,	2.04	1.79E-02
CAMK2	Calcium/calmodulin-dependent protein kinase II	2.04	2.70E-02
FAM36	Family with sequence similarity 36, member A	2.03	2.26E-03
C3orf10	Chromosome 3 open reading frame 10	2.02	3.48E-03
KRCC1	Lysine-rich coiled-coil 1	2.01	2.18E-02
DSEL	Dermatan sulfate epimerase-like	1.99	5.01E-03
ZNF50	Zinc finger protein 502	1.99	4.46E-02
GPR160	G protein-coupled receptor 160	1.99	2.72E-03
RAB28	RAB28 // RAB28, member RAS oncogene family	1.99	1.45E-02
AHNAK	AHNAK nucleoprotein 2	1.98	2.47E-02
RHEB	Ras homolog enriched in brain	1.96	6.64E-03
SGCB	Sarcoglycan, beta 43kDa dystrophin-associated	1.96	2.00E-04
SATB1	SATB homeobox 1	1.96	1.27E-02
APBB2	Amyloid beta (A4) precursor protein-binding, family B,	1.95	2.22E-05
KCNJ2	Potassium inwardly-rectifying channel, subfamily J,	1.95	2.04E-03
RAB23	RAB23, member RAS oncogene family	1.95	1.67E-02
MORN	MORN repeat containing 2	1.90	6.00E-03
PXK	PX domain containing serine/threonine kinase	1.90	1.77E-05
DUSP1	Dual specificity phosphatase 19	1.89	2.54E-03
ITGA1	Integrin, alpha 1	1.89	2.97E-02
BANF1	Barrier to autointegration factor 1	1.88	3.72E-02
ARHGE	Cdc42 guanine nucleotide exchange factor (GEF)	1.88	1.30E-02
JUN	Jun oncogene	1.88	3.90E-02
ZNF27	Zinc finger protein 271	1.88	5.42E-04
CCDC11	Coiled-coil domain containing 111	1.87	3.12E-03
GBE1	Glucan (1,4-alpha-), branching enzyme 1	1.87	1.06E-02
TAF1B	TATA box binding protein (TBP)-associated factor,	1.86	5.89E-03
C3orf62	Chromosome 3 open reading frame 62	1.86	2.14E-03
CALM2	Calmodulin 2	1.86	5.11E-03
SDCCAG	Serologically defined colon cancer antigen 10	1.86	3.49E-02
MAP1LC	Microtubule-associated protein 1 light chain 3	1.85	5.66E-03
GTF2H	General transcription factor IIH, polypeptide 5	1.85	1.80E-02
BBS7	Bardet-Biedl syndrome 7	1.85	2.19E-02
TTC35	Tetratricopeptide repeat domain 35	1.84	1.63E-02
LETM2	Leucine zipper-EF-hand containing transmembrane	1.84	1.28E-02
HIST1H	Histone cluster 1, H1b	1.84	5.43E-03
COPZ2	Coatomer protein complex, subunit zeta 2	1.84	1.50E-02
E2F6	E2F transcription factor 6	1.83	1.75E-02
ETFDH	Electron-transferring-flavoprotein	1.83	3.26E-02
HLTF	Helicase-like transcription factor	1.83	5.59E-03
ANKRD	Ankyrin repeat domain 50	1.82	6.77E-03
HIST1H2	Histone cluster 1, H2bb	1.81	1.63E-04
SNX24	Sorting nexin 24	1.81	1.42E-06
ANKRD	Ankyrin repeat domain 28	1.81	1.62E-02
C10orf5	Chromosome 10 open reading frame 55	1.81	8.04E-06
RNASE3	Ribonuclease, RNase A family, 3	1.80	3.74E-03
FERMT2	Fermitin family homolog 2	1.79	2.88E-
C1orf103	Chromosome 1 open reading frame 103	1.79	2.15E-
FLJ42957	FLJ42957 protein	1.79	3.04E-
MAP3K13	Mitogen-activated protein kinase kinase kinase 13	1.79	3.32E-
MT1X	Metallothionein 1X	1.79	3.48E-
ANO10	Anoctamin 10	1.78	5.74E-
CCDC99	Coiled-coil domain containing 99	1.78	1.05E-
MTBP	Mdm2-binding protein	1.77	3.43E-
ZNF714	Zinc finger protein 714	1.77	3.20E-
TOMM20	Translocase of outer mitochondrial membrane 20 homolog	1.77	3.09E-

SEPT7	Septin 7	1.77	9.59E-
ZFP112	Zinc finger protein 112 homolog	1.76	4.69E-
PAM	Peptidylglycine alpha-amidating monooxygenase	1.76	8.87E-
BTD	Biotinidase	1.75	7.34E-
UBXN2A	UBX domain protein 2A	1.75	2.45E-
CCR1	Chemokine (C-C motif) receptor 1	1.75	2.46E-
CT45A2	cancer/testis antigen family 45 member A2	1.75	1.78E-
SCML2	Sex comb on midleg-like 2	1.75	2.12E-
COPS4	COP9 constitutive photomorphogenic homolog subunit 4	1.74	8.49E-
AP3S1	Adaptor-related protein complex 3, sigma 1 subunit	1.74	4.73E-
AKT3	v-akt murine thymoma viral oncogene homolog 3	1.74	4.00E-

Table 2. Information of top 100 genes up-regulation in the CR cells. The average fold change and *p*-value were listed.

Gene Symbol	Gene Name	Fold change	*p*-Value
AQP3	Aquaporin 3	-3.99	3.04E-02
NPHS1	Nephrosis 1	-3.57	3.16E-02
LAPTM5	Lysosomal multispanning membrane protein 5	-3.26	3.88E-04
OR52I2	Olfactory receptor, family 52, subfamily I, member 2	-3.19	2.16E-02
FCER1A	Fc fragment of IgE, high affinity I, receptor for alpha	-3.07	2.46E-02
OR51B4	Olfactory receptor, family 51, subfamily B, member 4	-2.94	2.10E-02
TCEAL6	Transcription elongation factor A (SII)-like 6	-2.71	5.12E-03
KRTAP5-6	Keratin associated protein 5-6	-2.69	2.06E-02
FAM10A4	Family with sequence similarity 10, member A4	-2.68	5.27E-03
MTRF1L	Mitochondrial translational release factor 1-like	-2.63	1.11E-02
CYP24A1	Cytochrome P450, family 24, subfamily A, polypeptide	-2.59	2.12E-02
SHISA2	Shisa homolog 2	-2.52	1.24E-02
DKFZP434P211	POM121 membrane glycoprotein-like 1 pseudogene	-2.51	3.31E-02
SUSD2	Sushi domain containing 2	-2.48	1.04E-02
BMP7	Bone morphogenetic protein 7	-2.45	2.38E-03
MMP1	Matrix metallopeptidase 1	-2.35	1.81E-02
IFITM1	Interferon induced transmembrane protein 1	-2.28	4.11E-02
TSSK1B	Testis-specific serine kinase 1B	-2.27	7.02E-03
C10orf91	Chromosome 10 open reading frame 91	-2.25	2.14E-02
OR5M3	Olfactory receptor, family 5, subfamily M, member 3	-2.22	2.09E-02
FKBP9	FK506 binding protein 9, 63 kDa	-2.22	3.02E-02
BRD7P3	Bromodomain containing 7 pseudogene 3	-2.19	1.49E-02
SERP2	Stress-associated endoplasmic reticulum protein family	-2.19	4.59E-02
CYP2F1	Cytochrome P450, family 2, subfamily F, polypeptide 1	-2.18	6.68E-05
C1orf180	Chromosome 1 open reading frame 180	-2.12	7.81E-03
ATP5E	ATP synthase, H+ transporting, mitochondrial F1	-2.12	1.58E-03
HSD11B1	Hydroxysteroid (11-beta) dehydrogenase 1	-2.08	4.35E-02
WASH3P	WAS protein family homolog 3 pseudogene	-2.07	1.33E-02
DDIT4	DNA-damage-inducible transcript 4	-2.04	1.39E-04
EYA2	Eyes absent homolog 2 (Drosophila)	-2.04	1.07E-02
SYT16	Synaptotagmin XVI	-2.02	2.19E-02
C10orf58	Chromosome 10 open reading frame 58	-2.01	1.78E-02
NLRP2	NLR family, pyrin domain containing 2	-2.01	9.17E-06
PRSS35	Protease, serine, 35	-2.00	2.05E-03
CYGB	Cytoglobin	-1.98	3.69E-02
PDHA2	Pyruvate dehydrogenase (lipoamide) alpha 2	-1.98	8.61E-03
OR10H3	Olfactory receptor, family 10, subfamily H, member 3	-1.97	4.34E-02
S100A9	S100 calcium binding protein A9	-1.97	4.44E-02
LENEP	Lens epithelial protein	-1.97	2.85E-02
GPR119	G protein-coupled receptor 119	-1.96	4.45E-03
ROR2	Receptor tyrosine kinase-like orphan receptor 2	-1.95	1.83E-02
OR2C1	Olfactory receptor, family 2, subfamily C, member 1	-1.94	1.80E-03

IL17B	Interleukin 17B	-1.93	3.01E-02
METTL7A	Methyltransferase like 7A	-1.92	1.11E-02
TRGV3	T cell receptor gamma variable 3	-1.92	2.10E-02
ACP5	Acid phosphatase 5, tartrate resistant	-1.92	1.98E-02
IFNA21	Interferon, alpha 21	-1.91	1.72E-02
FBXO25	F-box protein 25	-1.90	6.34E-03
LEFTY1	Left-right determination factor 1	-1.90	1.85E-02
C6orf25	Chromosome 6 open reading frame 25	-1.89	6.85E-03
OR13J1	Olfactory receptor, family 13, subfamily J, member 1	-1.89	2.47E-02
KRTAP21-2	Keratin associated protein 21-2	-1.88	3.06E-02
CMPK2	Cytidine monophosphate (UMP-CMP) kinase 2, mitochondrial	-1.88	2.28E-02
OR2B6	Olfactory receptor, family 2, subfamily B, member 6	-1.88	4.48E-03
PAK6	p21 protein (Cdc42/Rac)-activated kinase 6	-1.84	1.18E-02
UBXN10	UBX domain protein 10	-1.83	2.12E-02
IL13	Interleukin 13	-1.83	4.78E-02
FAM136A	Family with sequence similarity 136, member A	-1.82	2.71E-02
BVES	Blood vessel epicardial substance	-1.82	1.31E-02
TNP1	Transition protein 1	-1.81	1.05E-02
DEFB123	Defensin, beta 123	-1.81	1.52E-02
KRT16	Keratin 16	-1.80	1.47E-03
NEU4	Sialidase 4	-1.80	3.66E-03
GPR148	G protein-coupled receptor 148	-1.80	1.64E-02
SLC6A10P	Solute carrier family 6	-1.79	2.01E-02
TNFSF9	Tumor necrosis factor (ligand) superfamily, member 9	-1.79	1.42E-04
CLCNKB	Chloride channel Kb	-1.79	1.13E-02
PSORS1C2	Psoriasis susceptibility 1 candidate 2	-1.78	1.35E-03
ABHD9	Abhydrolase domain containing 9	-1.78	4.70E-02
TMEM22	Transmembrane protein 22	-1.77	2.64E-02
PXT1	Peroxisomal, testis specific 1	-1.77	2.30E-03
SDR16C5	Short chain dehydrogenase reductase family 16C, member 5	-1.77	3.04E-05
OR10H1	Olfactory receptor, family 10, subfamily H, member 1	-1.76	1.08E-02
FGF23	Fibroblast growth factor 23	-1.76	4.11E-02
LSDP5	Lipid storage droplet protein 5	-1.76	3.66E-02
CDH29	Cadherin-like 29	-1.75	2.38E-08
SDR9C7	Short chain dehydrogenase reductase family 9C, member 7	-1.73	3.43E-02
AGR2	Anterior gradient homolog 2	-1.73	2.44E-02
THBD	Thrombomodulin	-1.73	5.89E-03
LOC554234	Ribosomal protein L36a pseudogene	-1.73	1.50E-02
C20orf173	Chromosome 20 open reading frame	-1.73	1.58E-04
LOC100129827	Hypothetical protein LOC100129827	-1.73	4.24E-03
UBL5	Ubiquitin-like 5	-1.73	3.34E-02
NKX6-2	NK6 homeobox 2	-1.73	2.16E-03
PROK2	Prokineticin 2	-1.73	3.77E-02
KIR2DL1	Killer cell immunoglobulin-like receptor, two domains, l	-1.73	6.42E-03
FGF22	Fibroblast growth factor 22	-1.73	3.05E-03
FOXO3	Forkhead box O3	-1.72	3.72E-02
PLA2G2D	Phospholipase A2, group IID	-1.72	1.91E-02
TLR9	Toll-like receptor 9	-1.72	1.61E-02
TRPV6	Transient receptor potential cation channel, subfamily V	-1.71	6.35E-03
ABAT	4-aminobutyrate aminotransferase	-1.70	1.57E-03
PLXNA4	Plexin A4	-1.70	2.95E-03
TAC4	Tachykinin 4 (hemokinin)	-1.70	7.13E-04
MYL7	Myosin, light chain 7, regulatory	-1.70	4.32E-03
IL1RN	Interleukin 1 receptor antagonist	-1.70	2.70E-05
RSPH10B	Radial spoke head 10 homolog B (Chlamydomonas)	-1.70	2.50E-02
OR4K17	Olfactory receptor, family 4, subfamily K, member 17	-1.70	7.54E-03
ZFP36	Zinc finger protein 36, C3H type, homolog	-1.70	4.76E-02
PLA2G12A	Phospholipase A2, group XIIA	-1.70	4.49E-02

Table 3. Gene ontology analysis of all CR-associated genes.

GOTERM_BP_DIRECT Term	Count (%)	p-value	Genes
**Up-regulated**			
GO:0006355 Regulation of transcription, DNA-templated	48 (14.08%)	7.89E-05	ERC1, MECOM, PRDM8, RALGAPA1, SATB1, SUPT4H1, TAF1B, TBPL1, ZNF625-ZNF20, APBB2, CLOCK, CCDC59, GTF2H5, HLTF, HINT1, IFT57, KLHL31, LRIF1, KAT7, NOCT, NCOA1, NUP35, SCML2, SNAPC1, TGFBRAP1, ZNF112, ZNF138, ZNF175, ZNF221, ZNF224, ZNF227, ZNF254, ZNF26, ZNF329, ZNF35, ZNF417, ZNF45, ZNF502, ZNF510 ZNF558, [illegible]
GO:0051301 Cell division	17 (4.99%)	5.05E-04	FBXO5, NEK1, NUF2, SPC25, SENP5, ANAPC11, ANAPC13, CSNK1A1, CDC7, KATNA1, MAPRE3, NCAPG, SKA1, [illegible]
GO:0006893 Golgi to plasma membrane transport	4 (1.17%)	3.07E-03	CCDC93, COMMD1, CHIC2, EXOC1
GO:0006468 Protein phosphorylation	18 (5.28%)	3.11E-03	AKT3, BMP2K, CDC42BPA, LOC400927-CSNK1E, NEK1, PXK, TBCK, CAMK2D, CSNK1A1, EIF2AK2, ICK,
GO:0015031 Protein transport	16 (4.69%)	4.56E-03	BBS4, BBS7, ERC1, RAB23, PAB7A, ATG7, COPZ2, CCDC93, COMMD1, ECT2, EXOC1, SCFD2, SNX24, SPIRE1, STXBP5,
GO:0007067 Mitotic nuclear division	12 (3.53%)	4.65E-03	FBXO5, NEK1, NUF2, SPC25, ANAPC11, ANAPC13, CSNK1A1, KATNA1, MAPRE3, SKA1, STAG1, TXNL4A
GO:0042384 Cilium assembly	8 (2.35%)	6.43E-03	ARL13B, BBS4, BBS7, NEK1, RAB23, ICK, IFT57, [illegible]
GO:0060271 Cilium morphogenesis	8 (2.35%)	1.04E-02	ARL13B, BBS4, BBS7, NEK1, RAB23, HSPB11, ICK, [illegible]
GO:0007264 Small GTPase mediated signal	11 (3.23%)	1.22E-02	ARL13B, DNAJC27, RAB23, RAB28, RAB7A, RAP1B, RALGPS2, RAP1GDS1, RHEB,
GO:0055119 Relaxation of cardiac muscl	3 (0.88%)	1.82E-02	CAMK2D, KCNJ2, SLC8A1
**Down-regulated**			
GO:0007275 Multicellular organism	23 (7.64%)	2.27E-06	ELF3, EYA2, TNFRSF4, WNT8A, CDX2, FOXA3, FZD10, GALR2, GGNBP1, HILS1, HOXB1, HIVEP3, LENEP, NOTCH1, PRRX2, ROR2, SFRP2, SHISA2, TSSK1B, [illegible]
GO:0007186 G-protein coupled receptor signaling	29 (9.63%)	3.06E-05	GNG8, GPBAR1, GPR119, GPR148, GPR174, F2R, FZD10, NMBR, OR1B1, OR13J1, OR2AE1, OR2B6, OR2C1, OR2T12, OF3A2, ORD4D10, ORD4K17, OR6F1, OR8I2, PROK2, PRLHR, RGS16, SFRP2, TAS2R3, TAS2R38, TAS2R40
GO:0001580 Detection of chemical stimulus involved in sensory perception of bitter taste	6 (1.99%)	1.89E-04	CST1, RTP2, RTP5, TAS2R3, TAS2R38, TAS2R40
GO:0050911 Detection of chemical stimulus involved in sensory perception of smell	17 (5.65%)	2.15E-04	OR1B1, OR10H1, OR10H3, OR13F1, OR13J1, OR2AE1, OR2B6, OR2C1, OR2T12, OR3A2, ORD10, OR4K17, OR5M3, OR51D1, OR52I2, OR6F1, OR8I2

GO:0048754 Branching morphogenesis of an epithelial tube	4 (1.33%)	3.49E-03	BMP7, MMP14, NOTCH1, MYCN
GO:0071456 Cellular response to hypoxia	6 (1.99%)	9.59E-03	MPL, NDRG1, BMP7, FOXO3, HYOU1, PTGIS
GO:0007588 Excretion	4 (1.33%)	1.33E-02	NPHS1, AQP3, CLCNKA, SCNN1B
GO:0006954 Inflammatory response	12 (3.99%)	1.37E-02	ELF3, NLRP2, S100A9, TNFRSF4, CHST4, F2R, IL13, IL17B, PLA2G2D, PROK2, TAC4, TLR9
GO:0071354 Cellular response to IL-6	3 (1.00%)	1.48E-02	CAMP, FGF23, PTGIS
GO:0030324 Lung development	5 (1.66)	1.93E-02	CEBPA, HSD11B1, MMP14, NOTCH1, MYNC

Table 4. List of primers used in the present study.

**Gene**	**Primer**
KITLG	F: TCAAGAGCCCAGAACCCAGGC<br>R: CCATGGCTGCCCAGTGTAGGC
SPC25	F: ACCTCCTGTCAGATGGCGGGA<br>R:GCAGTCAGTACTTCCAATTCCTGCT
KCNJ2	F: GCATGGTGGAAGCCACTGCCA<br>R: TGTCAGGGGGCGTGTCCGTA
HLTF	F: TCCACCATTCTGGGAACAGCGA<br>R: TTGGGGCGGGAGCTAGACAA
RAB23	F: CGAGGAGCCCAGGCTTGTGT<br>R: CTGACCGGAGTGACTTCCACCAG
MSRB3	F: GGAAGCGGCTAACACCCCTGC<br>R: CGGTGCCACTGCTATCCGCA
RHEB	F: ACTTGTAGACACAGCCGGGC<br>R: CTTGTGAAGCTGCCCCGTCC
ULBP1	F: GCCCATGGACACGGCAGAGG<br>R: TGTGCCTGGGGCCAGAGAGG
SATB1	F: CAGCACACCACCCAGCCGTC<br>R: GCACCGCGTTGCTCTCCTGT
TPMT	F: GCAAGACAAGTGGGTGAACGGCA<br>R: CTGTGTCCCCGGTCTGCAAACC
GPR160	F: ACGTCAAACAAACCAGCCCC<br>R: AGCTGTCAGGAAAACTGGATAATGC
EVI1	F: CAGGACTGGTGGGGCTGCAA<br>R: GGGGCTGGTCTTGGCTTGTGG
NDC80	F: CCTCTCCATGCAGGAGTTAAG A<br>R: GGTCTCGGGTCCTTGATTTTC T
NUF2	F: TGTTAAGCAATACAAACGCACAG<br>R: TGCCTTTTCAATACCGTCGTG
SPC24	F: GCCTTCCGCGACATAGAGG<br>R: CCTGCTCCTTCGCATTGAGA
